# Intraocular lens power calculation using standard formulas and ray tracing after DMEK in patients with Fuchs endothelial dystrophy

**DOI:** 10.1186/s12886-017-0547-7

**Published:** 2017-08-23

**Authors:** Maged Alnawaiseh, Lars Zumhagen, André Rosentreter, Nicole Eter

**Affiliations:** 1 Department of Ophthalmology, University of Münster Medical Center, Albert-Schweitzer-Campus 1, Building D15, 48149 Muenster, Germany; 2 Department of Ophthalmology, University of Würzburg, Würzburg, Germany

**Keywords:** Intraocular lens power calculation, DMEK, Ray tracing

## Abstract

**Background:**

The study presented here aims to optimize the accuracy of intraocular lens (IOL) power calculations in patients after DMEK by evaluation of the impact of the altered anterior/posterior corneal curvature relationship.

**Methods:**

Scheimpflug-based Oculus Pentacam imaging was performed after DMEK surgery for Fuchs endothelial dystrophy. The IOL power was calculated for all patients by ray tracing, aiming for postoperative emmetropia. We also performed the IOL calculation using four third-generation formulas (SRK-T, Hoffer-Q, Holladay-1 and Haigis). The residual refractions for the individual target IOL were compared and analyzed.

**Results:**

This retrospective study included 42 eyes of 33 patients (age 68.73 ± 10.11 years) after DMEK surgery. The differences between the expected residual refraction based on ray tracing and that predicted with the third-generation formulas were statistically significant (all formulas *p* < 0.001). The highest mean difference in the residual refraction between the target IOL measured by ray tracing and that calculated with third-generation formulas was found for the Haigis formula (0.90 ± 0.40 D), and the lowest mean difference for the SRK/T formula (0.73 ± 0.49 D).

**Conclusions:**

DMEK surgery induced a relevant change in the anterior to posterior corneal curvature relationship; this needs to be taken into account in the IOL power calculation to avoid hyperopic refractive surprises.

## Background

Fuchs endothelial dystrophy (FED) is a common disease requiring corneal transplantation. Patients with Fuchs endothelial dystrophy have been treated in the past with penetrating keratoplasty but over the last few years new surgical techniques of posterior lamellar keratoplasty have been introduced (Descemet stripping endothelial keratoplasty (DSEK), Descemet stripping automated endothelial keratoplasty (DSAEK) and Descemet membrane endothelial keratoplasty (DMEK)) [1, 2].

DMEK is the latest development in endothelial keratoplasty; with this procedure, damaged endothelial cells can be replaced using only donor Descemet’s membrane with endothelium [3]. This allows faster visual rehabilitation, improved visual acuity and improved corneal transparency [2–6]. Refractive change after DMEK has been described as slight [7, 8], Ham et al. showing a refractive change in spherical equivalent at 6 months post DMEK of +0.32 ± 1.01 diopter [7]. They propose that the hyperopic shift results from a reversal of a preceding myopic shift induced by stromal swelling in endothelial disease [7]. In a previous study, our group demonstrated a significant change in the refractive power of the posterior surface of the cornea while the anterior cornea remained nearly unchanged [9].

DMEK can be performed in phakic eyes with a clear lens without performing lens-surgery [10, 11]. When these patients develop clinically significant cataract post DMEK, the changes on the posterior surface induced by surgery may be relevant for IOL power calculation. In FED patients, who have additional, clinically significant cataract and need simultaneous phacoemulsification, DMEK can be performed together with cataract surgery in the same session [12]. A reliable IOL power calculation is also necessary in this situation.

The aim of this study is to evaluate the impact of the changed posterior corneal surface on IOL power calculation after DMEK.

## Methods

### Subjects and selection criteria

This retrospective study included 42 eyes of 33 patients who had undergone DMEK for FED at the Dept. of Ophthalmology, University of Münster Medical Center. The study was approved by the local ethics committee and adhered to the tenets of the Declaration of Helsinki.

Patients were examined using rotating Scheimpflug corneal and anterior segment tomography (Pentacam HR; Oculus, Wetzlar, Germany). All patients were examined after attaining refractive stability (minimum 3 months after surgery) [7] under the same conditions; the automatic release mode of the Pentacam was used to minimize examiner-induced errors and only Pentacam images of good quality (internal quality indicator of the Pentacam) were included. A skilled examiner performed the Pentacam imaging. Eyes with a history of trauma, corneal infection or intraocular inflammation, corneal scars, contact lens wear three weeks before measurement, clinically significant graft detachment or delayed corneal clearance were excluded.

### IOL power calculation

To allow accurate quantification of the impact of the changed posterior corneal surface after DMEK surgery on the calculation of IOL power, we used a standard axial length of 23.6 mm, an anterior chamber depth (ACD) of 3.0 mm and pupil entrance diameter of 2.5 mm for all patients. Optimized IOL constants were used, as published on the “User Group for Laser Interference Biometry” website: (http://ocusoft.de/ulib/index.htm).

The refractive errors for all patients were determined using the OKULIX ray tracing software and we used the same IOL (Carl Zeiss CT Asphina 409 M) with individual IOL power aimed at a level close to emmetropia. The OKULIX software was developed at the University of Mainz and has been described in detail in various publications [13–17]. The corneal power in this context was measured by ray tracing and is usually expressed as total corneal refractive power (TCRP). The TCRP is an internal Pentacam ray tracing calculation based on the anterior and posterior corneal radii and the pachymetry according to Snell’s law.

For the 3rd generation formulas, IOL power calculation was based on the sagittal corneal radii (mm) measured by the Pentacam (15°, 3.0-mm ring). The difference between the predicted postoperative residual-refraction (RR) of the IOL determined by ray tracing and the RR of the same IOL obtained using “3rd generation” IOL calculation formulas (namely the SRK-T, Hoffer-Q, Holladay-1, and Haigis formulas) [18–21], was calculated and analyzed. For each formula we used the author and formula specific recommendation to calculate the effective lens position.

### Statistical analysis

Data management was performed with Microsoft Excel 2010. IBM SPSS® Statistics 22 for Windows (IBM Corporation, Somers, NY, USA) was used for statistical analyses. The normality of the data distribution was tested using the Kolmogorov–Smirnov test and the data did not fit a normal distribution. Wilcoxon signed-rank tests were therefore used to compare differences in the RR obtained with IOL calculation formulas of the 3rd generation (SRK-T, Hoffer-Q, Holladay-1 and Haigis) and that measured by ray tracing, assuming left and right eyes of the same patient to be independent. Data are reported as mean ± standard deviation. The level of statistical significance was set at *p* ≤ 0.05.

## Results

Forty-two eyes of 33 patients (age 68.7 ± 10.1 years; 13 male, 20 female) were included in our study. Different parameters, including patient sex, age, laterality, and IOL power are presented in Table 1.

**Table 1:** Demographics of the Study Population

Subjects	33
Age (Year)	68.73 ± 10.11
Sex (F: M)	20:13
Laterality (r: l)	22:20
IOL Power (D)	20.43 ± 2.04

The mean IOL power calculated by Oculix was 20.4 ± 2.0 D. The difference in RR for the same IOL calculated by Oculix and by the 3rd generation formulas (SRK-T, Hoffer-Q, Holladay-1, and Haigis) was statistically significant (all formulas: *p* < 0.001). SRK/T formula showed the lowest mean difference in RR compared with Oculix (0.73 ± 0.49 D), while the highest mean difference was found for the Haigis formula (0.90 ± 0.40 D) (Table 2). In most cases, the differences in RR lay between 0.5 D and 1.0 D for all formulas (Table 3).

**Table 2:** The difference between the predicted postoperative residual-refraction (RR) of the IOL determined by ray tracing and of the same IOL calculated by “3rd generation” IOL calculation formulas

Formula	RR, (D)
SRKT	0.73 ± 0.49
Holladay 1	0.80 ± 0.43
Hoffer Q	0.80 ± 0.36
Haigis	0.90 ± 0.40

**Table 3:** Showing the distribution of the differences in predicted postoperative residual-refraction (between IOL determined by ray tracing and that calculated by “3rd generation” IOL calculation formulas)

	SRKT	Holladay 1	Hoffer Q	Haigis
<0.5 D	15 (36%)	10 (24%)	11 (26%)	4 (10%)
0.5 D ≤ RR ≤1 D	15 (36%)	21 (50%)	23 (55%)	23 (55%)
>1 D	12 (29%)	11 (26%)	8 (19%)	15 (36%)

## Discussion

DMEK is gaining in importance, and with advantages such as reduced immune reactions and impressive visual outcome, it may become the treatment of choice for corneal endothelial disease [2, 3, 5, 6, 12, 22, 23].

DMEK can be performed in patients with clinically significant cataract needing simultaneous cataract surgery and IOL implantation [12], but can also be carried out in phakic eyes with a clear lens without performing cataract surgery in the same sitting [10, 11]. Given the promise of near complete visual recovery and refractive stability over time with this procedure [7, 22], increasing interest is being focused on IOL power calculations for cataract surgery prior to, in the same setting or after DMEK. In clinical practice, hyperopic outcomes tend to occur after endothelial keratoplasty and in the past many surgeons have aimed to achieve a more myopic postoperative outcome and choose a refractive target of −0.75 to −1.00 D to reduce the chance of unintended hyperopic surprises [8, 11, 24].

Modern two-variable third-generation formulas such as SRK/T, Haigis, Holladay-1 and Hoffer-Q use the corneal power based on the anterior sagittal corneal radii, a fictitious refractive index and axial length for estimation of IOL power [25]. The cornea has two refracting surfaces, and for adequate IOL power calculation the anterior and the posterior corneal surfaces need to be taken into account [24]. Modern optical biometers such as the IOL-Master (Carl Zeiss Meditec, Jena, Germany) do not measure the posterior cornea directly, but instead estimate it using an assumed value for the refractive index of the cornea combined with measurements of the anterior corneal curvature [24]. Ray tracing (considering the anterior and posterior corneal surfaces as well as the pachymetry) achieved a median absolute error of 0.24 D; a considerable improvement of 9% to 14% over already excellent results obtained with classic formulas [26].

The changed corneal power and the hyperopic shift after triple DMEK have been described in various studies in the literature [7–9]. Both Schoenberg et al. and our group showed that the anterior curvature did not significantly change after DMEK [8, 9]. Morphological changes in posterior curvature of the cornea are to be expected after DMEK, because DMEK surgery acts mainly on the backside of the cornea. In contrast to third-generation formulas, the OKULIX ray tracing software uses measured anterior and posterior curvature as well as pachymetry to calculate corneal power.

There are many advantages in performing DMEK in phakic eyes without cataract surgery, including lower risk of retinal detachment and macula edema, retained ability to accommodate in younger FED patients, as well as a more precise lens calculation after postoperative de-swelling of the cornea [11]. However, to obtain this benefit, changes on the posterior surface of the cornea after DMEK must be taken into account. In other words, IOL power calculation should be based, not only on anterior corneal curvature but also on posterior corneal measurements.

This study has several limitations. First, it includes only pseudophakic eyes. Second, changes in corneal thickness were not taken into account. However, the impact of corneal thickness on calculations of corneal power is small [27]. Third, this study is also limited by the small sample size and retrospective design. Further research in a prospective setting with a larger patient population is needed to validate these findings. Studies with patients after phakic DMEK may provide a basis for development of a formula specifically designed for DMEK patients. However, such studies are very difficult to perform, because the number of cataract operations undertaken in patients after phakic DMEK is still low.

## Conclusions

In conclusion, this study demonstrates significant differences between IOL power calculations based on measurement of the anterior corneal curvature alone and those based on measurements of anterior and posterior corneal radii as well as pachymetry. These findings are based on overestimated keratometric power due to changes in the anterior/posterior corneal curvature relationship after DMEK. These results need to be taken into account on calculation of IOL power to avoid hyperopic refractive surprises similar to the well-documented hyperopic refractive surprises arising in patients after laser vision correction.

## Abbreviations

ACD: Anterior chamber depth
DMEK: Descemet membrane endothelial keratoplasty
DSAEK: Descemet stripping automated endothelial keratoplasty
DSEK: Descemet stripping endothelial keratoplasty
FED: Fuchs endothelial dystrophy
IOL: Intraocular lens
TCRP: Total corneal refractive power
